# Young stroke: An update on epidemiology, emerging risk factors, and future research directions

**DOI:** 10.1177/17474930251400524

**Published:** 2026-01-02

**Authors:** A Rasing, NA Hilkens, F-E de Leeuw

**Affiliations:** 1 Department of Neurology, Radboud University Medical Center, Nijmegen, The Netherlands; 2 Department of Neurology, Erasmus University Medical Center, Rotterdam, The Netherlands

**Keywords:** Ischemic stroke in young adults, incidence, risk factors, diagnostics, psychosocial sequelae

## Abstract

The incidence of ischemic stroke among young adults (aged 18–49 years) has risen over recent decades, particularly in high-income countries, contrasting with the decline seen in older populations. This trend represents a growing public health concern, as stroke at young age often leads to long-term psychosocial consequences and loss of productive life years. The increasing incidence may partly reflect a higher prevalence of traditional vascular risk factors, as well as the identification of non-traditional risk and trigger factors such as air pollution, sleep apnea, long working hours, vigorous exercise, and illicit drug use. Diagnostic evaluation in this young population is typically more extensive than in older patients, given the broad spectrum of potential underlying causes. A structured, multidisciplinary approach integrating vascular, hematologic, and cardiac assessment is essential for accurate etiological classification. Although functional outcomes are generally favorable, many young stroke survivors experience persistent psychosocial sequelae, including cognitive impairment, depression, anxiety, and fatigue, which significantly affect quality of life. Recurrence risk varies according to stroke etiology, with the lowest rates observed in patients with a cryptogenic stroke. These findings highlight the importance of more tailored secondary prevention strategies, as antiplatelet therapy is not without risks. Further research is needed to identify novel risk and trigger factors, refine prognostic tools, optimize secondary prevention, and develop interventions addressing the psychosocial recovery of young stroke survivors.

## Introduction

While the incidence of ischemic stroke among older adults has declined over recent decades, the opposite trend is observed among younger people. Adults aged 18 to 49 years are increasingly affected by ischemic stroke, with recent studies showing a consistent rise in incidence across both high- and low-income countries.^1–3^ This shift signals a growing public health concern, as stroke at young age may have lifelong consequences, potentially lasting for several decades. Its impact extends beyond the individual, by affecting family dynamics, social relationships and career development. In addition, it has profound societal impact due to loss of economic productivity during what are typically the most active years of life. Stroke at young age often requires a more extensive diagnostic workup due to its diversity of potential causes. Once the underlying etiology is identified, personalized treatment and follow-up are essential. Although functional outcomes are generally favorable, many patients face persistent symptoms such as fatigue, cognitive impairment, and psychological distress that hinder social participation and return to work.^4–8^ This more hidden burden highlights the importance of multidisciplinary care approaches that address both medical and psychosocial recovery.

In this issue of the *International Journal of Stroke* (*IJS*), we focus on ischemic stroke at young age, highlighting recent advances in the field and presenting novel research that expands our understanding of its etiology and outcomes. Furthermore, we address key challenges and future directions for new research.

## Epidemiology: rising stroke incidence among young people

Over the past two decades, advances in both primary and secondary prevention strategies, particularly in high-income countries (HICs), have contributed to a decline in the incidence of ischemic stroke among older adults (⩾55 years).^1,9,10^ However, this favorable trend does not extend to younger populations. As highlighted in the review by Nehme and Li^11^ in this issue of *IJS*, the incidence of ischemic stroke in individuals under the age of 50 is considerably rising in HICs. Although absolute incidences vary across studies, reflecting differences in methodology, diagnostic criteria and population characteristics, nearly all investigators report increasing incidences in younger compared to older age groups. Some more recent population-based studies suggest that this upward trend may have stabilized in some regions after 2010.

Large-scale studies from Brazil and China have reported increasing stroke incidence among young adults in low- and middle-income countries (LMICs) up to 2015; however, findings from other population-based studies (in LMICs) are conflicting, with some even suggesting a potential decline in incidence in more recent years.^2,3,12,13^

## Understanding the upward trend

This rising incidence underscores the need for a better understanding of the underlying drivers and risk factors. Identifying these contributors is crucial to developing more tailored and effective prevention strategies that may help reverse this trend. In HICs, part of the observed increase may be attributed to the improvement of diagnostic coding and hospital-based stroke registries.^14^

Advancements in neuroimaging, particularly magnetic resonance imaging (MRI) with diffusion-weighted imaging (DWI), have improved the accuracy of ischemic stroke diagnosis. MRI-DWI provides greater sensitivity for detecting ischemia compared to computed tomography (CT), potentially leading to higher detection rates and better differentiation from stroke mimics.^15,16^ This may partly explain the observed rise in stroke incidence among young adults.

As reported by Nehme and Li,^11^ and confirmed in the JACARANDA study by Gonzalez et al.,^17^ traditional vascular risk factors are common among young adults with ischemic stroke. Conditions such as hypertension, smoking, obesity, and hyperlipidemia are present in approximately 20–47% of patients within this age group.^18,19^ Diabetes and obesity, in particular, have shown a marked upward trend in prevalence. Yet, preventive strategies remain limited as current risk prediction models heavily rely on age, leaving younger individuals often undertreated. Still, rising incidence of ischemic stroke has also been observed in individuals without these traditional risk factors, suggesting that atherosclerosis alone cannot explain this trend. This points toward additional, yet partially unknown underlying mechanisms.

## Identification of novel risk factors

Beyond traditional vascular risk factors, several non-traditional contributors have been linked to the rising incidence of ischemic stroke among young adults. Environmental and lifestyle-related exposures, such as air pollution, have been associated with an elevated cardiovascular risk, particularly in individuals under 55 years of age.^20–22^ Moreover, factors like long working hours (>40 hours per week), low physical activity levels, and both acute and chronic psychosocial stress may further increase stroke risk at younger ages, as discussed by Nehme and Li^11^ in this issue. In contrast to low physical activity levels, vigorous exercise has also been identified as a potential trigger for stroke in young adults, as have sexual activity and flu-like diseases.^23^

The growing prevalence of illicit drug use among young people is a serious concern worldwide and contributes to an increased risk of stroke in younger populations. Particularly cocaine and amphetamines elevate stroke risk through mechanisms including hypertension, vasospasm, endothelial dysfunction, cardiomyopathy and, less commonly, cerebral vasculitis.^24,25^

A study by Deb et al.^26^ published in this issue identifies additional risk factors for early-onset stroke, including chronic kidney disease, cancer, sleep apnea, and bipolar disorder. The authors also report associations with non-white race, Hispanic ethnicity—possibly reflecting a greater burden of cardiovascular risk factors—and non-partnered status, further underscoring the multifactorial nature of stroke in young adults.

Regarding malignancy, patients with active cancer are at increased risk of ischemic stroke due to direct tumor effects, cancer-associated hypercoagulability, and treatment-related complications. Recent research reports a threefold to fivefold increased risk of cancer diagnosis within the first year after stroke in patients aged 15–49 compared to age-matched controls.^27^

In addition, a recent study indicates that pregnancy complications are linked to a higher risk of ischemic stroke in young women, especially strokes due to atherosclerosis. This emphasizes the elevated cardiovascular risk among women with such a history, even early in life.^28^

Although several new risk factors for stroke at young age have been identified over the past decade, further research is needed to validate these emerging risk factors and uncover others that have yet not been determined, paving the way for improved prevention and early intervention strategies.

## Defining stroke etiology: diagnostic approach

The diagnostic workup in young adults with ischemic stroke is generally more comprehensive than in older patients, reflecting the broad spectrum of potential underlying causes. However, as demonstrated by the JACARANDA study, standardized and comprehensive diagnostic protocols are not yet implemented globally, resulting in etiological subgroups that may be difficult to compare across populations.^17^

**Figure 1.**
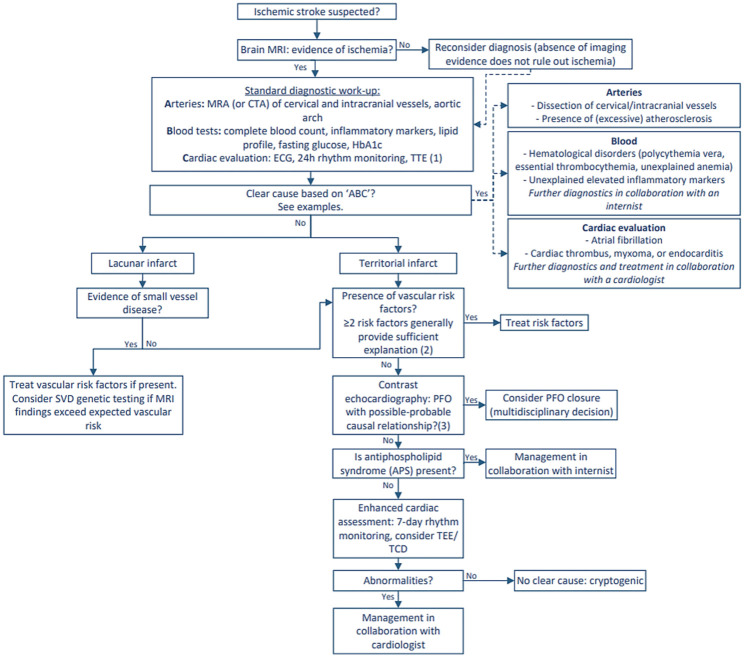
Diagnostic flowchart for young patients with ischemic stroke. ECG = electrocardiography, TTE = transthoracic echocardiography, SVD = small vessel disease TEE = transesophageal echocardiography, TCD = transcranial Doppler. (1): In young patients with ischemic stroke, a contrast-enhanced TTE is indicated unless acute phase diagnostics (“A” and “B”) already reveal a clear underlying cause, such as a dissection. (2): It is important to assess, on an individual basis (multidisciplinary), whether existing cardiovascular risk factors sufficiently explain the ischemic stroke. The number and severity of risk factors and the patient’s age should be considered. (3): To estimate the causal relationship, the RoPE score and PASCAL classification can be used.^29^ Rasing A, Hilkens NA, Leentjens J, et al. Diagnosis and treatment of ischemic stroke at a young age: a practical approach. *Tijdschrift voor Neurologie en Neurochirurgie* 2025; 126(7): 264–72.

The diagnostic workup can be approached systematically using an “ABC” framework, focusing on three key components: arteries, blood, and cardiac function. The accompanying flowchart (Figure 1) provides guidance for clinicians in practice.

Evaluation begins with neuroimaging, with MRI preferred due to its higher sensitivity in revealing ischemic patterns and its enhanced accuracy in detecting additional vascular brain damage compared to CT. Imaging of cervical (and intracranial) vessels is essential to identify atherosclerosis, arterial dissection or other arteriopathies that could potentially cause ischemic stroke.

Standard laboratory assessments include complete blood count (CBC), inflammatory markers, as well as lipid profile, fasting glucose and HbA1c to detect modifiable risk factors. Screening for antiphospholipid syndrome (APS) is recommended among selected patients, specifically those with a low cardiovascular risk profile. The updated diagnostic criteria from the American College of Rheumatology (ACR) and the European Alliance of Associations for Rheumatology (EULAR) highlight the importance of integrating clinical features with specific laboratory findings in the diagnostic evaluation of APS.^30^ Thus, APS testing should be considered only in selected cases with a low cardiovascular risk profile and no other determined cause of stroke.

Cardiac evaluation is a key component of the diagnostic process and includes electrocardiography and rhythm monitoring to detect arrhythmias such as atrial fibrillation, along with transthoracic echocardiography (TTE), to identify structural abnormalities including a patent foramen ovale (PFO) and atrial septal aneurysm (ASA). A contrast-enhanced TTE is recommended unless an underlying cause has already been identified during the acute diagnostic phase (e.g. arterial dissection). A PFO may be a cause of ischemic stroke in a subset of patients via the process of paradoxical embolism. However, given its high prevalence—approximately 25% in the general population—determining its causal relation might be challenging. In general, PFO closure should be considered only in patients without another evident cause of stroke. The decision should be made in a multidisciplinary setting, guided by risk stratification tools such as the Risk of Paradoxical Embolism (RoPE) score and PFO-Associated Stroke Causal Likelihood (PASCAL) classification, as the benefit of closure varies substantially between individuals.^29^

## Long-term prognosis

### Mortality

Mortality among young adults (18–49 years) with ischemic stroke is generally low, especially when compared to older populations. The highest risk of death occurs within the first year following the event (2.4%), with 10-year mortality rates ranging from 5.5% to 16%, approximately half of which are attributable to vascular causes.^31–33^ While several epidemiologic studies have reported declining mortality rates over time, others suggest that case fatality has plateaued.^34–36^ In this issue, Wang et al.^37^ present data on childhood and adolescent stroke (both ischemic stroke and intracerebral hemorrhage) mortality in China between 2005 and 2020, demonstrating an overall decline over time. Notably, males showed higher 15-year mortality and years of life lost (YLL) than females, and a rise in stroke mortality was observed in the 15–19 age group.

The general decrease in pediatric stroke case fatality may reflect greater understanding of pathophysiological mechanisms and improved acute and long-term stroke care. The observed sex disparity in pediatric stroke incidence and mortality, with higher rates in males, has led to the exploration of several underlying biological and environmental factors. Possible explanations are elevated testosterone levels that might contribute to the increased risk of ischemic stroke and higher incidence of trauma with subsequent risk for arterial dissection.^38–41^

### Recurrence risk

The risk of recurrent (ischemic) stroke in young adults has been examined in several large cohorts across different regions.^42–46^ In this issue of *IJS*, Kwok et al.^47^ present a comprehensive overview of long-term recurrence risk in this population through their systematic review and meta-analysis, pooling global data to provide robust estimates of recurrence. They report cumulative stroke recurrence rates of 3–7% within the first year, 11–13% at 5 years, and 14–20% at 10 years. Although these rates are lower than those observed in older populations, they still represent a substantial long-term burden of disease for young stroke survivors.^48–50^

Recurrence risk varies by stroke etiology, with the highest rates in patients with large artery atherosclerosis and the lowest in those with other determined etiology including arterial dissection, malignancy-associated thrombosis, APS, and Fabry disease, as observed in the analysis by Kwok et al. Grouping these diverse causes into a single category may lead to inaccurate prognostic interpretation, as they differ substantially in their outcomes.^51,52^ To accurately assess outcomes in these specific etiologies, a more refined classification model may be needed.

Notably, patients with undetermined etiology (according to the TOAST classification), including those with ⩾2 likely causes, negative or incomplete evaluation (with no details reported on the extent of the diagnostic workup), had a 12-month recurrence rate of approximately 13% in the pooled analyses by Kwok et al., which was lowest among all etiological subtypes. In contrast, lower rates have been reported in well-characterized European cohorts with 5-year follow-up (6–9%), likely reflecting more thorough diagnostic evaluations and stricter etiologic classifications.^42,43^

Hypertension, diabetes mellitus and a history of prior stroke or transient ischemic attack (TIA) emerged as predictors of recurrence, emphasizing the importance of early identification and optimal management of vascular risk factors. Although treatment of vascular risk factors in young adults often follows recommendations derived from studies in older populations, it is unclear whether these strategies can be directly extrapolated to younger patients. This highlights the importance of conducting research specifically focused on secondary prevention in this population.

### Functional outcome

Young adults who experience an ischemic stroke generally have favorable functional outcomes. Many show only mild neurological deficits on the National Institutes of Health Stroke Scale (NIHSS) or minimal disability on the modified Rankin Scale (mRS).^53^ In this issue of *IJS*, Pinter et al.^54^ report that nearly 75% of study participants maintained an excellent functional outcome (mRS 0–1, indicating no significant disability) at a median follow-up of 10.4 years. Nevertheless, despite generally good outcomes in terms of physical recovery, the long-term symptom burden remains high.^46,55^

### Psychosocial consequences

Beyond physical disability, young stroke survivors frequently experience long-lasting psychosocial symptoms that significantly impact daily functioning and quality of life. Approximately half of the young ischemic stroke patients develop cognitive impairment, often with little to no improvement over the first year post-stroke.^4,56^ In addition, fatigue, depression, and anxiety are also commonly reported among young stroke survivors.^5,6,57,58^ In this issue, Pinter et al.^54^ report on long-term non-motor outcomes after ischemic stroke or TIA at young age, with a median follow-up of 10.4 years. Cognitive impairment was present in 27.2%, fatigue in 27.6%, anxiety in 38%, and depression in 18.5%, with the latter three symptoms occurring more often in women. Notably, these symptoms were frequently reported even among patients with excellent functional recovery (mRS 0–1). The proportion of patients who successfully returned to work was 68%, consistent with previous findings.^8^ Cognitive impairment, poorer functional outcome, higher age, and lower educational level were associated with a reduced likelihood of returning to work.

These findings underscore the importance of recognizing and addressing psychosocial sequelae in young stroke survivors, to provide these patients adequate support and improve long-term outcomes.

## Future directions and current research

Future research in young-onset ischemic stroke should aim to identify additional trigger factors as well as develop prognostic tools to estimate individual recurrence risk and guide secondary prevention strategies.

Significant progress has been made in understanding the epidemiology and risk factors of ischemic stroke in young adults, yet several important areas warrant further investigation.^23,53,59^ As the incidence of stroke in this population is expected to rise, there is a need for greater awareness and advanced primary prevention strategies targeting known risk factors. In parallel, the identification of novel, potentially modifiable risk factors that may contribute to stroke but are not yet well recognized, remains essential. The SECRETO (Searching for Explanations for Cryptogenic Stroke in the Young: Revealing the Etiology, Triggers, and Outcome) study aims to address these topics, with a particular focus on patients with ischemic stroke of unknown cause (ClinicalTrials.gov ID: NCT01934725). By examining novel triggers, risk factors, genetic background, and long-term prognosis, this multicenter study strives to advance our understanding of the disease.^60^

An equally important but still underexplored research area is the management of persistent psychosocial symptoms, as effective interventions could improve prognosis and help patients regain their previous level of functioning.

Improving prognostic tools and optimizing long-term management according to stroke etiology remain important topics for future study as well, as outcomes vary significantly across etiologic subtypes. A particularly unresolved issue is the optimal duration of antiplatelet therapy in young patients with cryptogenic stroke. Findings from a Dutch cohort indicate that recurrence risk declines substantially after 2–5 years, raising questions about the need for long-term antiplatelet therapy, especially since one in five young patients experience a bleeding event requiring medical attention within 10 years after the initiation of antiplatelet therapy.^42,61^ To address this issue, the multicenter STOP trial (STopping Or continuing Platelet inhibitors after cryptogenic stroke at young age) has been launched in the Netherlands (ISRCTN96178713). This randomized clinical trial aims to determine whether discontinuation of antiplatelet therapy, at least 3 years after a cryptogenic stroke at young age, is non-inferior to continued treatment in preventing recurrent vascular events. Study completion is expected in 2032.

Another key issue regarding secondary prevention after ischemic stroke at young age is the optimal duration of antithrombotic therapy after PFO closure in cryptogenic stroke. Current European guidelines recommend dual antiplatelet therapy for 1 to 6 months after PFO closure, followed by single antiplatelet therapy for at least 5 years.^62^ However, it is well established that the recurrence rate after cryptogenic stroke and PFO closure is low. A recently published study from an Austrian cohort reports a recurrence rate of 0.38 per 100 patient-years over a median follow-up of 10 years, which aligns with earlier studies, though those had shorter follow-up durations.^63–66^ As earlier mentioned, prolonged antithrombotic use increases the risk of bleeding.^61,66^ To evaluate whether the benefit of prolonged antithrombotic therapy outweighs the bleeding risks, the HALTI trial (Discontinuation of Antithrombotic Treatment Following Patent Foramen Ovale Closure in Young Patients With Cryptogenic Stroke) was initiated in 2020 to investigate whether antithrombotic therapy can safely be stopped 12 months after PFO closure in young patients with cryptogenic stroke (ClinicalTrials.gov ID: NCT04475510). The study is currently still recruiting participants, with the study completion estimated in 2032.

## Conclusion

The rising incidence of ischemic stroke in young adults is an alarming trend that calls for deeper insights into its underlying trigger factors and mechanisms. Although substantial progress has been made in identifying novel risk factors, future research should aim to uncover additional contributors that lead to an increased risk of cerebrovascular events in this age group. Given the considerable long-term impact on psychosocial functioning, healthcare systems must be organized to ensure adequate follow-up and treatment for persistent post-stroke symptoms. Furthermore, as recurrence risk differs by stroke etiology, there is need for individualized prognostic tools to guide more risk-adapted secondary prevention strategies.

This issue of *IJS* presents some interesting work that contributes to a better understanding of the epidemiology, risk factors, and prognosis of stroke in the young.
